# Ex vivo expanded human regulatory T cells modify neuroinflammation in a preclinical model of Alzheimer’s disease

**DOI:** 10.1186/s40478-022-01447-z

**Published:** 2022-09-30

**Authors:** Alireza Faridar, Matthew Vasquez, Aaron D. Thome, Zheng Yin, Hui Xuan, Jing Hong Wang, Shixiang Wen, Xuping Li, Jason R. Thonhoff, Weihua Zhao, Hong Zhao, David R. Beers, Stephen T. C. Wong, Joseph C. Masdeu, Stanley H. Appel

**Affiliations:** 1 Stanley H. Appel Department of Neurology, Houston Methodist Research Institute, 6560 Fannin Street, Suite ST-802, Houston, TX 77030 USA; 2 T. T. and W. F. Chao Center for BRAIN, Houston Methodist Hospital, Houston, TX USA; 3 Systems Medicine and Bioengineering Department, Houston Methodist Cancer Center, Houston, TX USA

**Keywords:** Alzheimer’s disease, Regulatory T cells, Inflammation, Adaptive immune system, Microglia, Amyloid pathology

## Abstract

**Background:**

Regulatory T cells (Tregs) play a neuroprotective role by suppressing microglia and macrophage-mediated inflammation and modulating adaptive immune reactions. We previously documented that Treg immunomodulatory mechanisms are compromised in Alzheimer’s disease (AD). Ex vivo expansion of Tregs restores and amplifies their immunosuppressive functions in vitro. A key question is whether adoptive transfer of ex vivo expanded human Tregs can suppress neuroinflammation and amyloid pathology in a preclinical mouse model.

**Methods:**

An immunodeficient mouse model of AD was generated by backcrossing the 5xFAD onto Rag2 knockout mice (5xFAD-Rag2KO). Human Tregs were expanded ex vivo for 24 days and administered to 5xFAD-Rag2KO. Changes in amyloid burden, microglia characteristics and reactive astrocytes were evaluated using ELISA and confocal microscopy. NanoString Mouse AD multiplex gene expression analysis was applied to explore the impact of ex vivo expanded Tregs on the neuroinflammation transcriptome.

**Results:**

Elimination of mature B and T lymphocytes and natural killer cells in 5xFAD-Rag2KO mice was associated with upregulation of 95 inflammation genes and amplified number of reactive microglia within the dentate gyrus. Administration of ex vivo expanded Tregs reduced amyloid burden and reactive glial cells in the dentate gyrus and frontal cortex of 5xFAD-Rag2KO mice. Interrogation of inflammation gene expression documented down-regulation of pro-inflammatory cytokines (*IL1A&B, IL6*), complement cascade (*C1qa, C1qb, C1qc, C4a/b*), toll-like receptors (*Tlr3, Tlr4 and Tlr7*) and microglial activations markers (*CD14, Tyrobp,Trem2*) following Treg administration.

**Conclusions:**

Ex vivo expanded Tregs with amplified immunomodulatory function, suppressed neuroinflammation and alleviated AD pathology in vivo. Our results provide preclinical evidences for Treg cell therapy as a potential treatment strategy in AD.

**Supplementary Information:**

The online version contains supplementary material available at 10.1186/s40478-022-01447-z.

## Background

The discovery of risk genes involved in inflammation signaling [1–3], indicates that inflammation is critical for the onset and progression of Alzheimer’s disease (AD). In AD transgenic mouse models, microglia is one of the initial responders to amyloid-β plaque deposits [4–6]. Activation of microglial pattern recognition toll-like receptors (TLRs) and intracellular NLRP3 inflammasomes induces tau hyperphosphorylation and aggregation [7–11]. Subsequent release of truncated phosphorylated tau also enhances immune cell activation, promoting the release of inflammatory mediators and a self-propagating cascade of synaptic dysfunction, neuronal injury, and cell death [11–14]. The structural integrity of the blood–brain barrier is also impaired in the presence of AD pathology [15–17], thereby permitting substantial crosstalk between the central nervous system (CNS) and peripheral immune system. Thus, circulating immune cells might reflect and contribute to AD pathogenesis [18–20]. Regulatory T cells (Tregs) are a subset of T cells that play a neuroprotective role by suppressing inflammation in the blood and brain [21]. We have previously documented that Treg immunomodulatory mechanisms are compromised in AD patients. As a consequence, there is activation of systemic cytotoxic immune cells and upregulation of pro-inflammatory mediators [22]. Accumulating preclinical and clinical evidences suggest Tregs as a modifiable therapeutic target. Ex vivo expansion of dysfunctional Tregs in AD individuals not only restored but enhanced their immunosuppressive function [22]. Restoration of Tregs is currently being translated into cell therapy for neurodegenerative disorders. Recently, our group has conducted a first-in-human Phase I trial of expanded autologous Treg infusions in Amyotrophic Lateral Sclerosis (ALS) [23]. The study demonstrated safety and potential benefit of this treatment strategy, and a Phase II double blind, placebo-controlled study is currently underway. The promising therapeutic potential of autologous infusions of expanded Tregs in ALS raised the question as to whether a similar strategy would be of value in AD. In the current study, we investigated the impact of ex vivo expanded human Tregs on neuroinflammation and Aβ pathogenesis in a preclinical AD system.

The widely used 5xFAD mice model recapitulates early and aggressive features of AD pathology with sustained microglial activation, increased inflammation markers and abundant deposition of plaques at early ages [24, 25]. To evaluate the role of the adaptive immune system on AD pathology, a mouse model of AD was generated by backcrossing the 5xFAD mouse onto a Rag2 Il2rγ^–/–^ double knockout mice (5xFAD-Rag2KO). The resulting mice lack T cells, B cells and natural killer (NK) cells. This Rag2KO mouse model is an appropriate host for xenogeneic human cell engraftment with minimal host versus graft reaction [26–31]. Ex vivo expanded human Tregs with remarkably enhanced immunosuppressive function were adoptively transferred to the immunodeficient 5xFAD-Rag2KO mice. Following peripheral administration, Tregs were detectable in the frontal cortex and dentate gyrus. They effectively reduced numbers of reactive microglia and astrocytes, suppressed neuroinflammation transcriptome and alleviated amyloid burden.

## Methods

### Generation of a Rag2 Il2rγ^–/–^ knock out immune-deficient AD mouse model

All animal protocols were approved by the Methodist Research Institute's Institutional Animal Care and Use Committee in compliance with National Institutes of Health guidelines. Rag2 Il2rγ^–/–^ double knock out mice were initially bred with purebred 5xFAD and strain-matched wild-type C57BL/6 mice to generate immune-deficient 5xFAD-Rag2KO and strain-matched WT-Rag2KO mice. The presence or absence of the Rag2 gene was determined by PCR using 250 ng of tail DNA and Eppendorf TaqDNA polymerase according to the manufacturer’s instructions. The following primers were used: Rag A) 5′-GGGAGGACACTCACTTGC-CAG-3′ and Rag B) 5′-AGTCAGGAGTCTCCATCTCAC-3′ and Neo C) 5′-CGGCGG-GAGAACCTGCGTGCAA-3′. Homozygotic mice will have one 350 bp band. Heterozygotic mice will have 350 and 263 bp bands. Wild-type mice will have one 263 bp band. Therefore, six groups of mice were characterized in this study including WT-5xFAD, 5xFAD-Rag2KO, Treg-treated 5xFAD-Rag2KO, WT-WT, WT-Rag2KO and Treg-treated WT-Rag2KO mice. There were total 10 mice per group, sex balanced (5 female & 5 male).

### Ex vivo expansion of human Tregs and passive transfer to mice

Human CD4^+^CD25^high^ T lymphocytes were isolated from peripheral blood of a healthy subject using the Regulatory T Cell Isolation Kit (Miltenyi Biotec) according to the manufacturer’s instructions. Tregs were suspended at a concentration of 1 × 10^6^ cells/ml in media containing 100 nM of rapamycin (Miltenyi Biotec), 500 IU/ml IL-2 (Miltenyi Biotec) and DynabeadsTM Human Treg Expander (GibcoTM) at a 4:1 bead-to-cell ratio for 8 days (First stimulation). At day 8, beads were removed, and cells were resuspended in a culture medium containing 100 U/mL IL-2 and 100 nM of rapamycin for 8 days. On day 16, Tregs were restimulated by adding Dynabeads expansion beads at a 1:1 bead-to-cell ratio for further 8 days. After the second stimulation, Tregs were harvested and washed on day 24. The Treg immunophenotype and suppressive function were assayed prior to injection to mice, as described previously [22]. Based on the data obtained from similar studies in infusing Treg into preclinical mice model [32–34], and considering temporal expression profile of CNS inflammatory genes in 5xFAD mice [24], the current study was designed to administer 1 × 10^6^ ex vivo expanded Tregs, suspended in a 200 μl of phosphate-buffered saline (PBS), into 5-month-old 5xFAD-Rag2KO and WT-Rag2KO mice via tail vein injections. This treatment was repeated every month for a total of 5 months and the mice were sacrificed at age 10 months.

### RNA sample preparation and transcriptome analysis

Using Trizol reagent, followed by Direct-zol RNA MiniPrep Kit (Zymo Research), messenger RNA was extracted from medial temporal cortex and frontal cortex of mice. For mouse neuroinflammation panel analysis, 770 transcripts were quantified with the NanoString nCounter multiplexed target platform (www.nanostring.com). nCounts of mRNA transcripts were normalized using the geometric means of 10 housekeeping genes (Csnk2a2, Ccdc127, Xpnpep1, Lars, Supt7l, Tada2b, Aars, Mto1, Tbp, and Fam104a).

### Protein extraction and ELISA assay

The right hemisphere samples were homogenized in a 2% SDS lysis buffer (SDS, NaCl 150 mM and Triton™ 1%) containing phosphatase (Pierce) and protease (Roche) inhibitors. After centrifugation (60 min, 100,000×*g*, 4 °C), the supernatant was collected (SDS extract) and the protein concentration was quantified. 70% formic acid in water was added to the pellet and the supernatant was collected after sonication and centrifugation (FA extract). Soluble (SDS extract) and insoluble (FA extract) Aβ40 and Aβ42 were measured using Amyloid beta Human ELISA Kit (Invitrogen).

### Immunofluorescence staining

Splenocytes were isolated from spleens for flow cytometric analysis. Antibodies against the following surface markers were provided by: CD3 FITC (eBioscience™), CD4 PE (eBioscience™), CD8a Alexa Fluor 700 (eBioscience™), CD161 APC (eBioscience™) and CD19 PE-Cy5 (eBioscience™). Dead cells were stained by LIVE/DEAD® Fixable Blue Dead Cell Stain Kit (Life Technology). For immunohistochemical brain analyses, the left cerebral hemisphere was dissected and post-fixed in 4% paraformaldehyde in 0.1 M PBS for 2 days. Brains were cryoprotected by incubation in a 30% sucrose/0.1 M PBS solution. Sagittal brain sections were cut on a freezing microtome (Leica) and collected serially. Immunohistochemistry was performed on free-floating microtome-cut Sects. (10 μm in thickness). Sections were incubated with different antibodies: anti-mouse Iba1 (Polyclonal, 1:1000 Wako), anti-mouse CD68 (Clone FA-11, 1:200; BioRad), β-Amyloid (Clone 6E10, 1:1000; BioLegend), β-Amyloid 1–42 (polyclonal, 1:100; Millipore), anti-mouse GFAP (Clone GA-5, 1:100, Novus Biological) anti-human CD3 (Clone: CD3-12, 1:100, abcam) and anti-human Foxp3 (Clone 236A/E7, 1:100, Invitrogen). Appropriate secondary antibodies (Alexa Fluor 488, 594, or 647; Invitrogen) were used followed by incubation with DAPI.

### Confocal image quantification

After immunofluorescence staining, 2D single plane image were captured using a Nikon A1 laser scanning confocal microscope. The system uses a galvanometer scanner with a 20 × Plan Apo objective, and a pinhole set to 1.2 Airy Unit. Laser power, numeric gain and magnification were kept constant between animals to avoid potential technical artefacts. NIS Elements Version 5.11.01 was used to quantify mean intensity of fluorescence, number of immunoreactive cells, and size of plaques. The region of interest to analyze was done across the field of view of the whole image acquired. Since each image was 1024 × 1024 pixels with a resolution at 0.63um/pixel, we measured the amount of immunoreactive cells in a 416,179.81 um2 area of frontal cortex or dentate gyrus. All absolute quantifications were performed at × 20 magnification. The DAPI mask of detected nuclei was used as a reference to generate the count of positive microglial cells. Any mask of detected microglial signal that had overlapping signal with the DAPI mask of detected nuclei were considered positive microglial cells and a new detected objects mask was created. All other detected DAPI nuclei were excluded from this new mask. In evaluating number of microglia that were surrounding plaques (within 20-μm radius of the 6E10-positive plaques), two separate FITC masks of detected amyloid beta plaques were generated. One mask of detected amyloid beta plaques was used to gain an accurate measurement of amyloid beta plaque area and count. The other mask of detected amyloid beta plaques was a copy of the first amyloid beta plaque mask but with the implementation of dilating the mask to increase the diameter up to an additional 20 µm. The new DAPI nuclei mask of positive microglial cells, mentioned earlier, was used to determine the quantity of microglial cells associated with the dilated mask of detected amyloid beta plaques by overlaying both masks. After analysis of the masks of detected objects, statistical data was retrieved. The counts of positive glial cells, count of positive glial cells associated with amyloid beta plaques, the total detected area of amyloid beta plaque, the summation of all detected amyloid beta plaque signal intensity, and total count of detected amyloid beta plaque were analyzed.

### Statistical analysis

In this exploratory study, we applied resource equation method with projected attrition of 20, to determine the sample size in each group, as described previously [35, 36]. All experiments in this study were blinded and randomized by blocks of animals. The experimental design and handling of mice were identical across experiments. The vehicle group were treated with the same volume of vehicle via tail vein injections. The analyses, including immunohistochemistry staining, ELISA and transcriptome analysis were performed by blinded independent investigators. Statistical analysis was performed using Prism 7.0 (GraphPad Software). The significance of group comparisons was tested using paired or unpaired student’s t-test (for two groups) or one-way ANOVA (for more than two groups). Data were expressed as Mean ± SEM and p values less than 0.05 were considered significant. For transcriptome analysis, nSolver software was used for background subtraction and normalization of data. Statistical analysis on the normalized expression profiles, including one-way ANOVA and multiple comparison using Tukey’s range test, were carried out using the Statistics and Machine Learning Toolbox in MATLAB R2020a. Volcano plots of differential expressed genes data were plotted using GraphPad Prism. Gene enrichment analysis was performed using Ingenuity pathway analysis (IPA). 

## Results

### Treg administration alleviates amyloid burden in immunodeficient AD mice

**Fig. 1:**
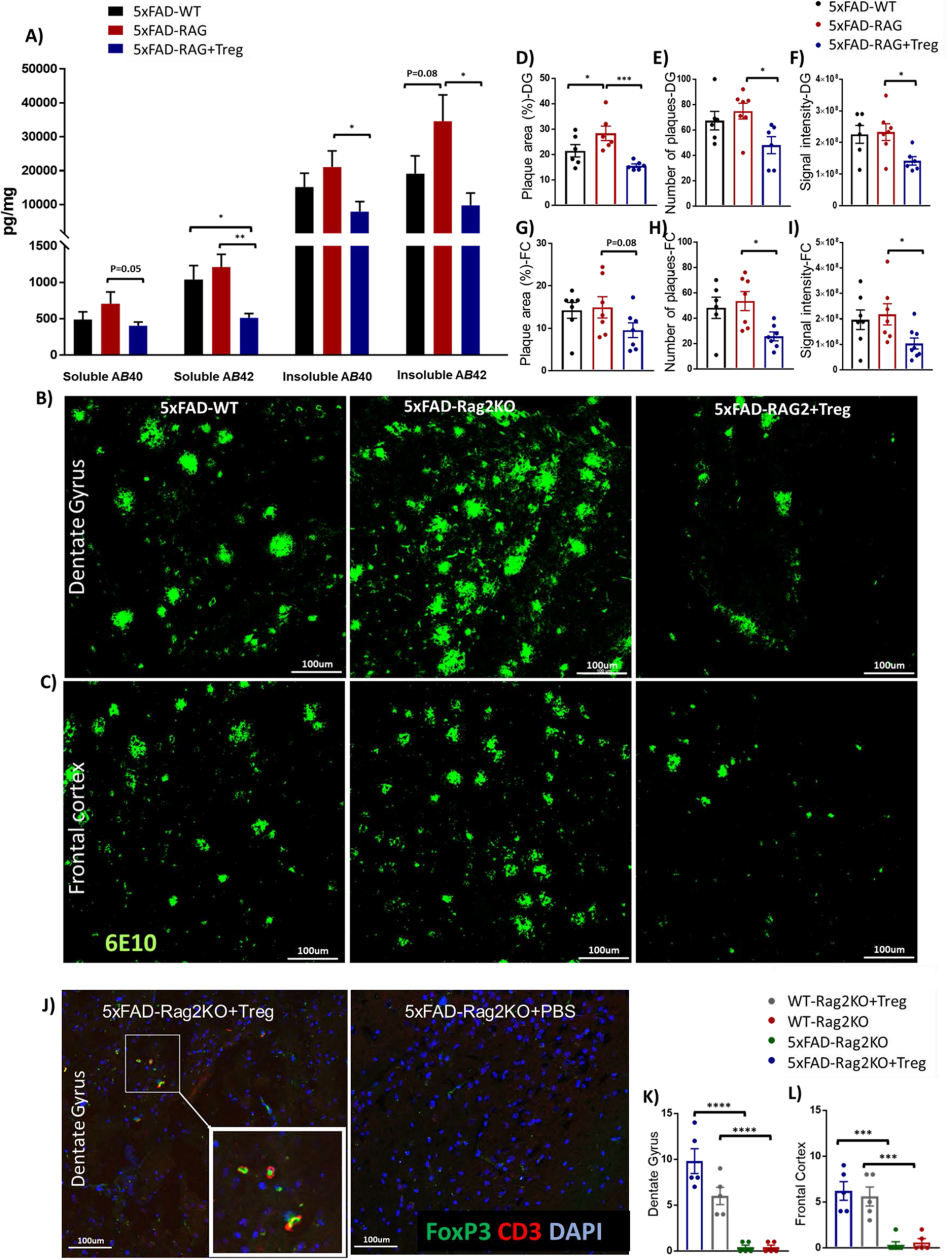
Treg treatment suppress Amyloid pathology. **A** ELISA quantification of mice brain homogenates shows a trend toward an increased levels of SDS-soluble and formic acid treated insoluble Aβ40 and Aβ42 in 5xFAD-Rag2KO, compared to 5xFAD-WT. Ex vivo expanded Treg administration decreased both soluble and insoluble Aβ40 and Aβ42 burden in 5xFAD-Rag2KO (10-month-old mice, 10 per group; sex-balanced). **B**, **C** Representative images of Aβ immunostaining (6E10) of the dentate gyrus (DG) and frontal cortex (FC) in 5xFAD-WT, 5xFAD-Rag2KO and Treg-treated 5xFAD-Rag2KO. **D**–**I** Quantification of the 6E10-positive amyloid aggregates in the DG and FC. The percentage of area covered by Aβ in the DG were increased in 5xFAD-Rag2KO compared to 5xFAD-WT. Number of plaques and their signal intensity were comparable between 5xFAD-Rag2KO and 5xFAD-WT in both DG and FC. Ex vivo expanded Treg administration reduced area covered by plaque, number of plaques and their signal intensity in both DG and FC of 5xFAD-Rag2KO mice (n = 6–7 per group). **J** Immunostaining of Tregs (CD3 in red, Foxp3 in green) and cell nuclei (DAPI in blue) in the DG of 10-month-old 5xFAD-Rag2KO mice treated with human Tregs or Phosphate-buffered saline (PBS). **K**, **L** Quantification of number of CD3^+^Foxp3^+^ Tregs in the DG and FC of 4 groups of mice including Treg treated 5xFAD-Rag2KO, Treg treated WT-Rag2KO, PBS treated 5xFAD-Rag2KO and also PBS treated WT-Rag2KO mice. Numbers shown as averages ± SEM with one-way ANOVA. P-values are *p < 0.05, **p < 0.01, ***p < 0.001, ****p < 0.0001. Scale bar, 100 μm

We generated 5xFAD-Rag2KO and WT-Rag2KO mice by backcrossing the 5xFAD and C57BL/6 J mice onto a Rag2 Il2rγ^–/–^ double knock out background. We have documented lack of adaptive immune system including CD4 and CD8 T cells, B cells and NK cells in transgenic Rag2KO mice (Additional file 1: Fig. S1A). These cells are the key immune components involved in xenogeneic cell rejection. Strain-matched immunocompetent AD and wild-type (WT) mice, termed 5xFAD-WT and WT-WT respectively, were also developed. CD4^+^CD25^high^Tregs were isolated from peripheral blood of a healthy human subject and expanded ex vivo. After two rounds of stimulation, the number of Tregs was increased 56 times within 24 days (Additional file 1: Fig. S1B). The increase in number of Tregs following ex vivo expansion, was associated with amplification in mean fluorescence intensities (MFI) of immunomodulatory markers including Foxp3, CD25, PD1 and CD73 (Additional file 1: Fig. S1C–F). We also evaluated the immunosuppressive function of Tregs at baseline and following 8 and 24 days of ex vivo expansion. The suppressive function of Treg on corresponding CD4^+^CD25^−^ T responder (Tresp) proliferation and iPSC-derived pro-inflammatory myeloid cells (M1) cytokine expressions were significantly enhanced following ex vivo expansion (Additional file 1: Fig. S1G–J). Tregs vs. vehicle were adoptively transferred into 5xFAD-Rag2KO and WT-Rag2KO mice and the presence of Tregs in the murine brain was evaluated after 2 weeks with co-labeling against CD3 and Foxp3 as described previously [32]. Human Tregs were detected and quantified in the DG and FC of 5xFAD-Rag2KO mice following peripheral administration, suggesting that these human cells survived in immunocompromised mice and distributed into the CNS (Fig. 1J–L, Additional file 1: Fig. S2).

Highly sensitive multiplex ELISA was performed to quantify the burden of SDS-soluble and formic acid (FA) treated insoluble Aβ within the brains of immunocompetent 5xFAD-WT, immunodeficient 5xFAD-Rag2KO and Treg-treated 5xFAD-Rag2KO mice (n = 10/group; sex-balanced). In the absence of the adaptive immune system, there was a trend toward increased levels of Aβ40 and Aβ42 species in 5xFAD-Rag2KO, compared to 5xFAD-WT. Treg administration effectively reduced the levels of both soluble and insoluble Aβ40 and Aβ42 in 5xFAD-Rag2KO mice, compared to the untreated group. Levels of soluble Aβ42 in Treg-treated 5xFAD-Rag2KO were even lower than in the immunocompetent 5xFAD-WT group (Fig. 1A). Soluble and insoluble Aβ40 and Aβ42 were undetectable in WT-WT and WT-Rag2ko mice. Sections of dentate gyrus (DG) and frontal cortex (FC) were also immunolabeled with 6E10 antibody to assess plaque load, using confocal microscopy (Fig. 1B, C). As expected, 5xFAD-WT mice, at age 10-month, showed highly abundant plaques in the DG and FC. In comparison between 5xFAD-WT and 5xFAD-Rag2KO, while the number of plaques (Fig. 1E, H) and their signal intensities (Fig. 1F, I) were comparable, the absence of T, B and NK cells in 5xFAD-Rag2KO, further increased the percentage of area covered by Aβ in the DG (Fig. 1D). Following Treg administration, the total plaque area (Fig. 1D, G) and the number of plaques (Fig. 1E, H) within the DG and FC of 5xFAD-Rag2KO mice were alleviated. The signal intensity of plaques was also lowered in the Treg-treated AD group, suggesting reduced plaque compaction following Treg administration (Fig. 1F, I).

### Treg administration modifies glial cells in AD mice

**Fig. 2:**
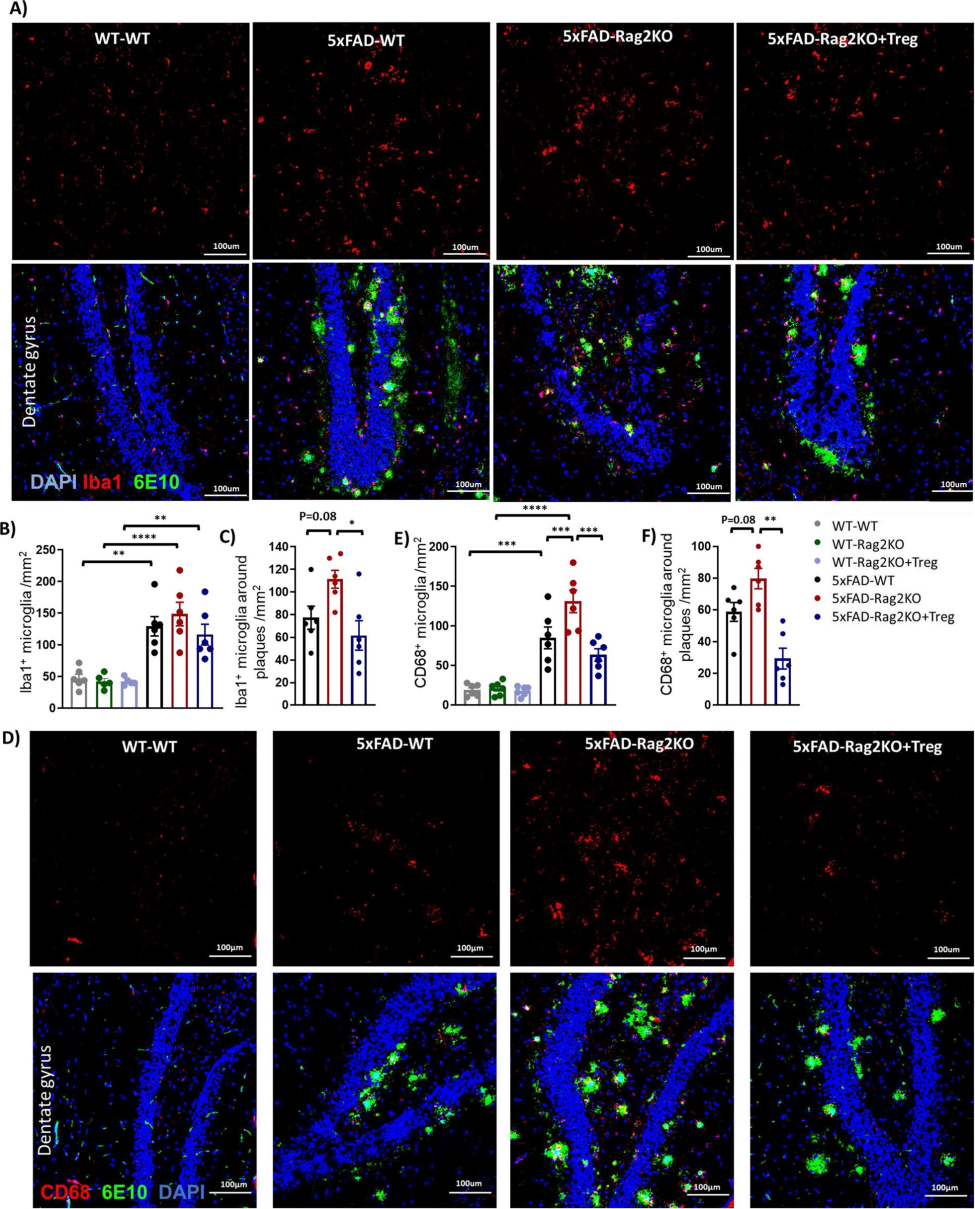
Treg administration reduces number of activated microglia in the dentate gyrus. **A** Representative images of Iba1 positive-microglia (red) and 6E10-positive Aβ plaques (green) in the dentate gyrus (DG) of WT-WT, 5xFAD-WT, 5xFAD-Rag2KO and Treg-treated 5xFAD-Rag2KO (n = 6 per group, sex balanced). **B** Quantification of the number of Iba^+^ microglia in the DG; while number of Iba1^+^microglia was increased in 5xFAD-WT mice, compared to WT-WT, lack of adaptive immune system in 5xFAD-Rag2KO and subsequent Treg administration had no effect on the number of Iba1^+^ microglia. **C** Quantification of the number of Iba1^+^ microglia within 20 μm of the plaque surface; Treg administration reduced number of plaque- associated Iba1^+^ microglia. **D** Representative images of CD68-positive activated microglia (red) and 6E10-positive Aβ plaques (green) in the dentate gyrus. Quantification of CD68-positive activated microglia in the DG (**E**) and CD68-positive microglia within 20 μm of the plaque surface (**F**), revealed decreased number of total and plaque-associated CD68^+^ microglia in 5xFAD-Rag2KO following Treg administration. Numbers shown as averages ± SEM with one-way ANOVA. *p < 0.05, **p < 0.01, ***p < 0.001 and ****p < 0.0001. Scale bar, 100 μm

Compared to WT, the 10-month-old 5xFAD-WT mice showed 2–3 folds increases in the number of Iba1^+^ microglia in the DG and the FC. The total number of Iba1^+^ microglia was comparable between 5xFAD-WT and 5xFAD-Rag2KO and remained unaltered after Treg administration (Fig. 2A, B, Additional file 1: Fig. S3A, B). In evaluating number of microglia that were surrounding plaques (within 20-μm radius of the 6E10-positive plaques), there was a trend toward increased level of plaque associated Iba1^+^ microglia in the DG of 5xFAD-Rag2KO mice, compared to 5xFAD-WT (Fig. 2C). Treg administration significantly reduced plaque associated Iba1^+^ microglia in the DG (Fig. 2C). In the next step, the number of activated microglia was assayed in the DG and FC, using CD68 staining (Fig. 2D and Additional file 1: Fig. S3D). The number of CD68^+^ microglia within the DG was increased following elimination of T, B and NK cells in 5xFAD-Rag2KO mice, compared to 5xFAD-WT (Fig. 2E). Treg treatment significantly reduced total and plaque associated CD68^+^microglia in both DG (Fig. 2E, F) and FC of 5xFAD-Rag2KO (Additional file 1: Fig. S3E, F).

**Fig. 3:**
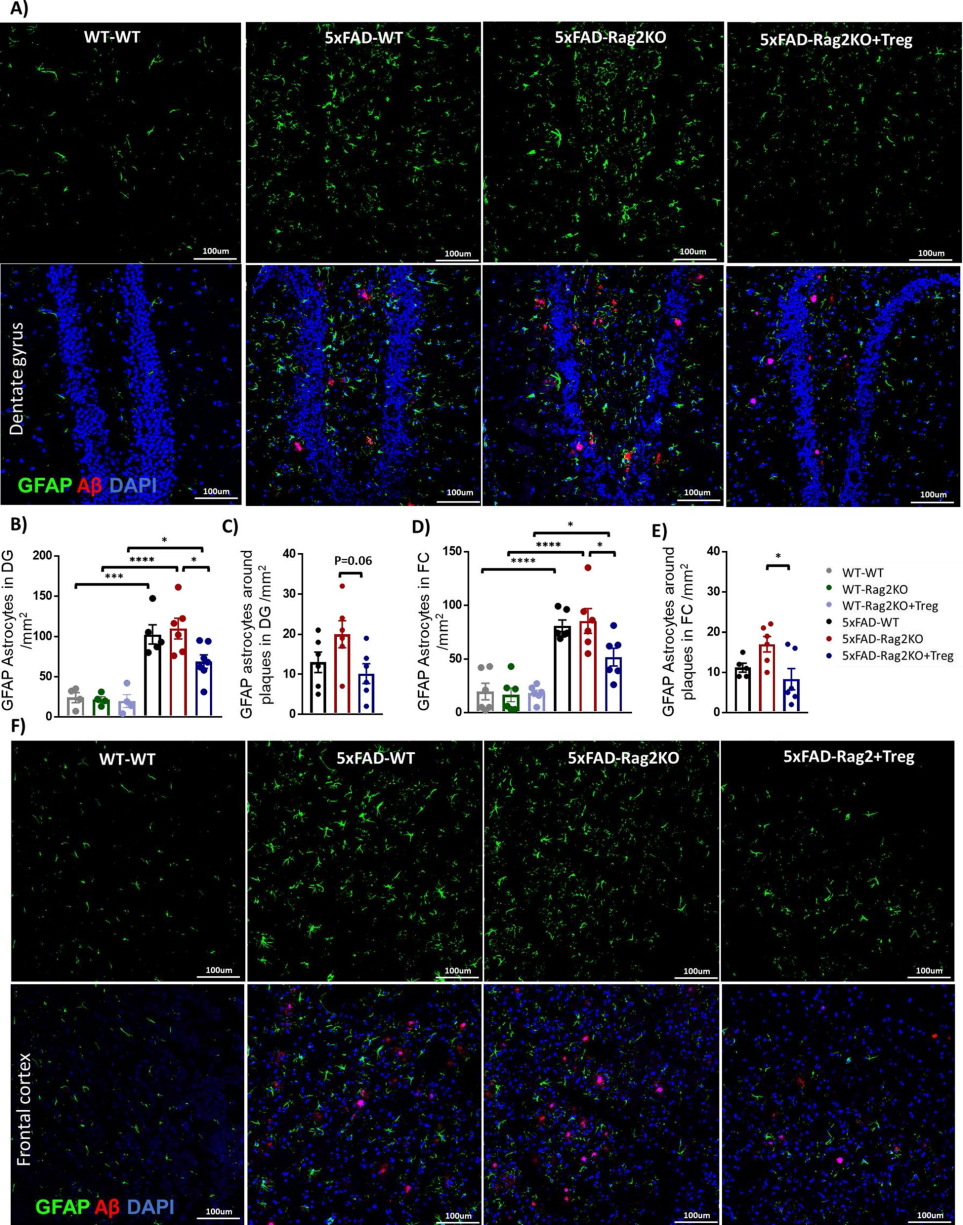
Treg administration reduces number of reactive astrocytes. Representative images of GFAP positive-reactive astrocytes (green) and Aβ 1–42 positive plaques (red) in the dentate gyrus (DG) (**A**) and frontal cortex (FC) (**F**) of WT-WT, 5xFAD-WT, 5xFAD-Rag2KO and Treg-treated 5xFAD-Rag2KO (n = 6 per group, sex balanced). **B**–**E** Quantification of the number of GFAP^+^ reactive astrocytes in the DG and FC; number of GFAP^+^ reactive astrocytes was increased in 5xFAD-WT mice, compared to WT-WT. Lack of adaptive immune system in 5xFAD-Rag2KO had no effect on the number of GFAP^+^ reactive astrocytes, compared to 5xFAD-WT. Decreased number of total and plaque-associated GFAP^+^ reactive astrocytes in 5xFAD-Rag2KO were noted following Treg administration. Numbers shown as averages ± SEM with one-way ANOVA. *p < 0.05, **p < 0.01, ***p < 0.001 and ****p < 0.0001. Scale bar, 100 μm

In the next step, reactive astrocytes were evaluated in the DG and FC, using GFAP staining. There were major differences in astrocyte state in AD relative to WT group, as number of GFAP^+^ reactive astrocytes in 5xFAD mice were about 4 folds higher than WT mice in the DG and FC (Fig. 3A, F). While number of GFAP^+^ reactive astrocytes were comparable between 5xFAD-WT and 5xFAD-Rag2 KO group, Treg treatment significantly alleviated number of GFAP^+^ reactive astrocytes in both DG and FC of 5xFAD-Rag2 KO mice (Fig. 3B, D). In evaluating plaque associated reactive astrocytes, there was a trend toward increased level of GFAP^+^ reactive astrocytes that were located within 50 um of amyloid plaques in the DG and FC of 5xFAD-Rag2KO mice compared to 5xFAD-WT. Treg treatment suppressed plaque associated GFAP^+^ reactive astrocytes in both DG and FC (Fig. 3C, E).

### Identification of differentially expressed inflammation genes following adaptive immune system modification

**Fig. 4:**
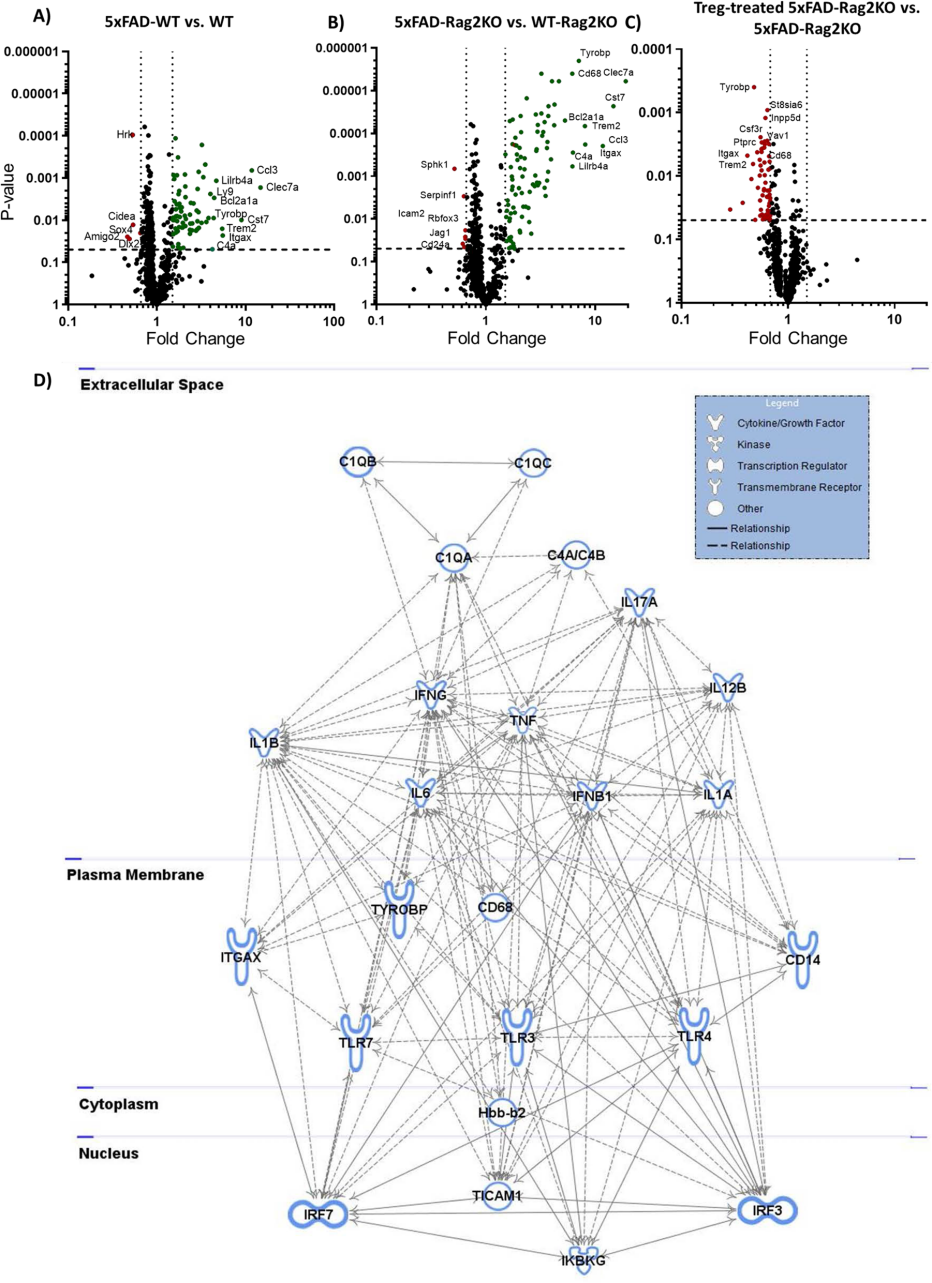
Modification of inflammation network following Treg administration. Volcano Plots showing fold changes vs. p-values of nCounter Mouse Neuroinflammation panel in **A** 5xFAD-WT vs. WT-WT**;** 73 immune related genes were upregulated (green dots) and 5 genes were down regulated (blue dots) in 5xFAD-WT (n = 4 in each groups). **B** Compared with WT-Rag2KO, 95 genes were upregulated (green dots) and 7 genes were down-regulated (blue dots) in 5xFAD-Rag2KO. The 10 most upregulated genes and all down-regulated genes are labeled in the figures. **C** Treg-treated 5xFAD-Rag2KO vs. 5xFAD-Rag2KO; the pathologic upregulation of inflammation related genes in 5xFAD-Rag2KO were modified following Treg administration. **D** The network representation and subcellular assignment of the enriched pathway in Treg-treated 5xFAD-Rag2KO vs. untreated 5xFAD-Rag2KO. The enriched network were centered in down-regulation of pro-inflammatory cytokines (*IL1A&B, IL6, Tnfa, IFNγ*), complement activation (*C1qa, C1qb, C1qc, C4a/b*), toll like receptors (*Tlr3, Tlr4* and *Tlr7*), myeloid activation markers (*CD14, Tyrobp,Trem2*) and intra-nuclear binding motifs of interferon-regulatory factors (*IRF3* and *IRF7*)

NanoString gene expression panel of 770 mouse immune factors was applied in the extracted RNA from the hippocampus of WT-WT, WT-Rag2KO, 5xFAD-WT, 5xFAD-Rag2KO and Treg-treated 5xFAD-Rag2KO. Comparing of 5xFAD-WT with WT-WT mice, 73 immune related genes were upregulated (Fold change (FC) > 1.5, *p* < 0.05) and only 4 genes were downregulated (FC < 0.66, *p* < 0.05) in AD mice (Fig. 4A). Genes implicated in microglial/macrophage activation [*Clec7a*: FC = 14.7, *Ccl3*: FC = 11.7, *Cst7*: FC = 9.0, *Trem2*: FC = 5.4, *Tyrobp*: FC = 4.36, *Cd68:* FC = 3.6] as well as complement activation (*Itgax*: FC = 5.5, *C4a*: FC = 4.2) were most highly upregulated in 10-month-old 5xFAD-WT mice (Fig. 4A), supporting the role of neuroinflammation in AD pathogenesis. Comparing 5xFAD-Rag2KO with WT-Rag2KO, 95 immune genes were upregulated and 7 genes were down-regulated in immunocompromised AD mice. Lack of T, B and NK cells in 5xFAD-Rag2KO further strengthened the pathological overexpression of microglial/macrophage (*Clec7a*: FC = 19.0, *Cst7:* FC = 14.6, CCL3: FC = 11.3, *Trem2:* FC = 8.0, *Tyrobp:* FC = 7.0 and *CD68:* FC = 6.1) and complement activation (*Itgax:* FC = 8.1, *C4a:* FC = 6.2) markers (Fig. 4B). Finally, we evaluated the impact of ex vivo expanded human Tregs on modulating neuroinflammation transcriptome. 71 out of 99 upregulated genes in 5xFAD-Rag2KO, had opposite direction of fold changes following Treg treatment**,** which suggests Treg treatment is pulling the expression profile of these immune genes away from 5xFAD-Rag2KO and towards WT-Rag2KO (Fig. 4C). Applying Ingenuity’s pathway analysis, we interrogated these genes with respect to enriched network and subcellular compartments. The enriched network was centered in extracellular complements activation (*C1qa, C1qb, C1qc, C4a/b*), pro-inflammatory cytokine networks (*IL1A&B, IL6, Tnfa, Ifnγ*), membrane binding receptors of toll like receptors (*Tlr3, Tlr4* and *Tlr7*) and myeloid activation markers (*Cd14, Tyrobp,Trem2, Cd68*). In addition, nuclear localized binding motifs of the interferon-regulatory factors (*Irf3 and Irf7*) which provides further evidence for modification of interferon response-related genes following Treg treatment (Fig. 4D). In order to evaluate the finding of pathway integrative analysis beyond the hippocampus, 10 enriched genes in hippocampal analysis, including complement activation markers (*C1qa, C1qb, C1qc*), pro-inflammatory cytokines (*IL1B, IL6, TNFa*), microglial activation markers (*Tyrobp, Trem2*) and transmembrane protein genes of *Tlr3* and *Itgax* were analyzed in the extracted RNA from the frontal cortex, using quantitative real-time PCR. 8 out of 10 selected genes were significantly upregulated in 5xFAD-RAG2 KO mice, and subsequently down-regulated or had a trend toward reduced expressions following Treg administration (Additional file 1: Fig. S4).

## Discussion

Our study characterized the impact of ex vivo expanded human Tregs in a preclinical AD model. Following peripheral administration, Tregs were detectable in the central nervous system of immunodeficient AD mice and reduced reactive glial cells and amyloid burden. Similar to our finding, systemic Treg expansion in transgenic AD mice models, including low dose IL-2 administration, modified neuroinflammation and enhanced neuroprotection against AD pathology [32, 37, 38]. However, Tregs might have dichotomous effects on the neurodegenerative process by obstructing a selective gateway for immune cell trafficking to the CNS, reducing the recruitment of immunoregulatory cells from the periphery to the CNS particularly in juvenile mice [39]. We believe that in older AD mice, in the presence of AD pathology, or in the clinical setting of Alzheimer’s disease, the structural integrity of the blood–brain barrier has already broken down and peripheral immune cells would migrate through the activated endothelium by various mechanisms.

We initially generated immunocompromised AD mice model with lack of adaptive immune system. Genetic ablation of T cells, B cells and NK cells in AD mice, further amplified the expression of pathologic inflammatory genes involved in complement cascade and microglial activation. The activation of complement cascade and pro-inflammatory microglia can lead to synaptic dysfunction, neuronal death and inhibition of neurogenesis [40–42]. The upregulation of these neuroinflammatory networks and increased number of activated glial cells in this study were associated with increased percentage of area covered by Aβ, as reported previously [43]. In contrast to our finding and Marsh et al. study that applied Rag2 Il2rγ^–/–^ double knock out in 5xFAD mice (Lack of B, T and NKC), Späni et al. used single knock out Rag2 PSAPP transgenic mice (NKC sufficient) with contradicting reduction in CNS amyloid burden [44]. Together, these results indicate that NK cells might play a considerable role in microglial activation and amyloid pathology.

Accumulating preclinical and clinical evidences suggest that adaptive transfer of ex vivo expanded Tregs is a novel therapeutic strategy to modulate chronic inflammation in neurodegenerative processes [23]. Following ex vivo expansion, the immunophenotype and suppressive function of ex vivo expanded Tregs were substantially restored and amplified. In the current study, we investigated the modulatory effect of ex vivo expanded human Treg with amplified immunosuppressive function on AD pathology in a preclinical AD mice model. The 5xFAD-Rag2KO mice lack T cells, B cells and natural killer cells, key immune components involved in xenogeneic cell rejection and are suitable for engraftment of human cells without preconditioning [26–31, 45–47]. Following peripheral administration, Tregs were detectable in the dentate gyrus and frontal cortex of mice. This finding is consistent with previous study that has shown survival of human Treg in immunocompromised mice for at least 40 days after administration [33] and also supported transmigration of peripheral Tregs into the CNS [38, 48, 49]. To monitor immunophenotypic persistence of adoptively transferred Tregs in vivo, in a separate experiment, we isolated lymphocytes from the blood and spleen, 3 and 7 days following peripheral infusion of human Tregs, to reevaluate their characteristics with flow cytometry. However, the number of events appeared to be very low with unreliable immunophenotypic analysis (unpublished data). In this regard, further studies are still required to assess the detailed distribution and immunophenotypic persistence of adoptively transferred Treg following peripheral administration. Other potential limitations in interpretation of our finding is the unknown specificity of human Tregs on modifying neuroinflammation rather than nonspecific effect of human cells in a preclinical AD mice model and also unknown extend of cross-reactivity between human Tregs immunomodulatory markers and mice immune system, as the presence of mice MHC rather than human MHC might limit the function of human Treg. However, the amplified expression of immunomodulatory markers including CTLA4, CD73 and PD1 on the surface of polyclonally expanded Treg cell clearly can contribute to the suppressive mechanism of action and modulating glial cells that is independent of HLA expression.

Microglia are one of the initial responders to amyloid-β plaque deposits [4–6]. While, low concentrations of Aβ can be taken up by microglia and concentrated into acidic vesicles. Excessive accumulation of Aβ within microglial lysosomes might induce cellular death, potentially contributing to plaque expansion through the release of Aβ aggregates at the site of microglial death [50]. In this context, other studies identified roles of activated inflammatory microglia in initiating and expanding plaque pathogenesis rather than phagocytosis and removal [50–53]. In our study, Treg administration to 5xFAD-Rag2KO mice, did not alter overall microgliosis but did reduce the number of reactivate microglia surrounding the plaques.

Astroglia are thought to be the most prevalent cell type in the brain [54, 55]. Aβ oligomers and inflammatory mediators transform resting astrocytes to hyperplastic and hypertrophic GFAP-high disease associated astrocytes which secrete further pro-inflammatory cytokines and upregulate APP expression [56–58]. In a recent study, adaptive transfer of Tregs into Rag2KO mice suppressed neurotoxic astrogliosis in the chronic phase of stroke [59]. Our data expands this finding to AD pathology as Treg therapy effectively alleviated total and plaque associated GFAP^+^ reactive astrocytes in both dentate gyrus and frontal cortex.

Finally, the pathologic upregulation of inflammation genes in 5xFAD-Rag2KO was modified following Treg administration. Using Ingenuity Pathway Analysis, we noted that the enriched pathways were mainly associated with down-regulation of pro-inflammatory cytokines (*IL1A&B, IL6*), complement cascade (*C1qa, C1qb, C4a/b*), toll like receptors (*Tlr3, Tlr4* and *Tlr7*) and microglial activations markers (*CD14, Tyrobp,Trem2*). These canonical signaling pathways are mainly reported as part of “microglial pro-inflammatory responses” to toxic Aβ [60]. We propose that reduced number of plaque-associated glial cells and suppression of pro-inflammatory signaling pathways within these cells following Treg therapy have attenuated the contribution of these toxic glial cells in AD pathology and mitigated amyloid burden. However, further investigation is required to evaluate Treg potential effects on AD glial heterogeneity and single cell immunophenotypic shifting.

## Conclusion

In this study, ex vivo expanded human Tregs were adaptively transferred to immunodeficient 5xFAD-Rag2KO mice. Following peripheral administration, Tregs were detectable in the central nervous system, suppressed neuroinflammation and substantially alleviated amyloid pathogenesis. These promising preclinical findings provide a rationale for enhancing Treg immunomodulatory function with infusions of ex vivo expanded Tregs or in vivo expansion of endogenous Tregs in patients with Alzheimer’s disease.

## Supplementary Information

**Additional file 1.** Supplementary Figure 1: Characteristics of ex vivo expanded Tregs. Supplementary Figure 2: The presence of Tregs in the frontal cortex following peripheral administration. Supplementary Figure 4: Inflammation gene expressions in the frontal cortex.

## Data Availability

The datasets supporting the conclusions of this article are included within the article and its additional supplementary file. The other raw and analysed datasets generated during the study are available for research purposes from the corresponding author on reasonable request.

## Abbreviations

AD: Alzheimer’s disease
ALS: Amyotrophic lateral sclerosis
CNS: Central nervous system
FA: Formic acid
FC: Fold change
IPA: Ingenuity pathway analysis
iPCS: Induced pluripotent stem cells
MFI: Mean Fluorescence Intensities
NK: Natural killer
Treg: Regulatory T cells
Tresp: T responders
TLR: Toll-like receptors
WT: Wild type
