# Supplementary material for: Ex vivo expanded human regulatory T cells modify neuroinflammation in a preclinical model of Alzheimer’s disease
Source: Acta Neuropathol Commun. 2022 Sep 30;10:144. doi: 10.1186/s40478-022-01447-z (PMC9524037; doi:10.1186/s40478-022-01447-z)
Supplement: Supplementary file 1 — Additional file 1. Supplementary Figure 1: Characteristics of ex vivo expanded Tregs. Supplementary Figure 2: The presence of Tregs in the frontal cortex following peripheral administration. Supplementary Figure 4: Inflammation gene expressions in the frontal cortex. [file 40478_2022_1447_MOESM1_ESM.docx]

**Supplementary Material:**

**Supplementary figure 1-** **(A)** Immunophenotypic characterization of mice: Splenocytes were isolated from the spleen of WT-WT, 5xFAD-WT, WT-Rag2 KO and 5xFAD-Rag2 ko mice. CD3^+^CD4^+^ T cells, CD3^+^CD8^+^T cell, CD3^-^CD19^+^ B cell, CD3^-^CD161^+^NK cell populations (% of total lymphocytes) were analyzed with flow cytometry. Lack of adaptive immune cell (B, T and NK cells) were documented in 5xFAD-Rag2KO and WT-Rag2KO mice. The percentage of T cells, B cells and NK cells were not significantly different between 5xFAD-WT and WT-WT. **(B)** Column separated human Tregs were stimulated in vitro by adding CD3/CD28 expansion beads at Day 0 (D0) and Day 16 (D16). Ex vivo expansion of human Tregs increased their numbers by 56 times at day 24 (D24). Mean Fluorescence Intensities (MFI) of Foxp3 **(C)**, CD25 **(D)**, PD1 **(E)** and CD73 **(F)** were progressively amplified in Treg population at D8 and D24 of ex vivo expansion (each experiment repeated three times). **(G)** Suppression (%) of Tregs on Tresp proliferation (1:1 ratio) were enhanced at D8 and D24, compared to D0. **(H-J)** Tregs were added to iPSC-derived pro-inflammatory macrophages (M1) and relative suppression (%) of Tregs on M1-pro-inflammatory cytokines transcripts was measured. Expanded Tregs at D8 and D24 displayed an enhanced capacity to suppress M1-derived pro-inflammatory cytokine transcripts (each experiment repeated three times). Numbers shown as averages ± SEM with one-way ANOVA. P-values are *p < 0.05, **p< 0.01 and ***p< 0.001.


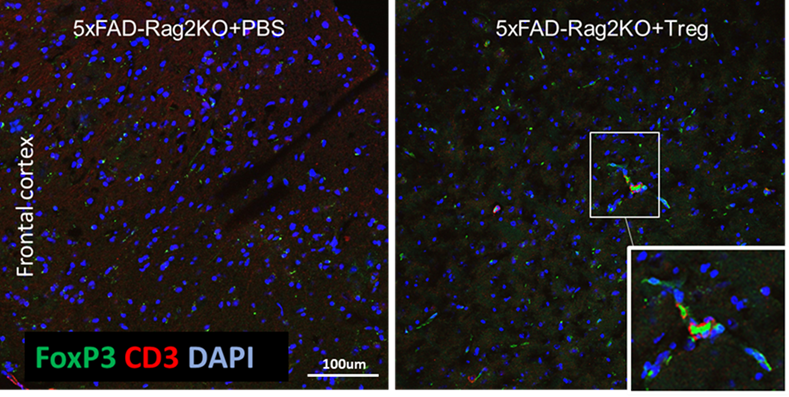


**Supplementary figure 2-** Immunostaining of Tregs (CD3 in red, Foxp3 in green) and cell nuclei (DAPI in blue) in the frontal cortex of 10-month-old 5xFAD-Rag2KO mice treated with Phosphate-buffered saline (PBS) or human Tregs.


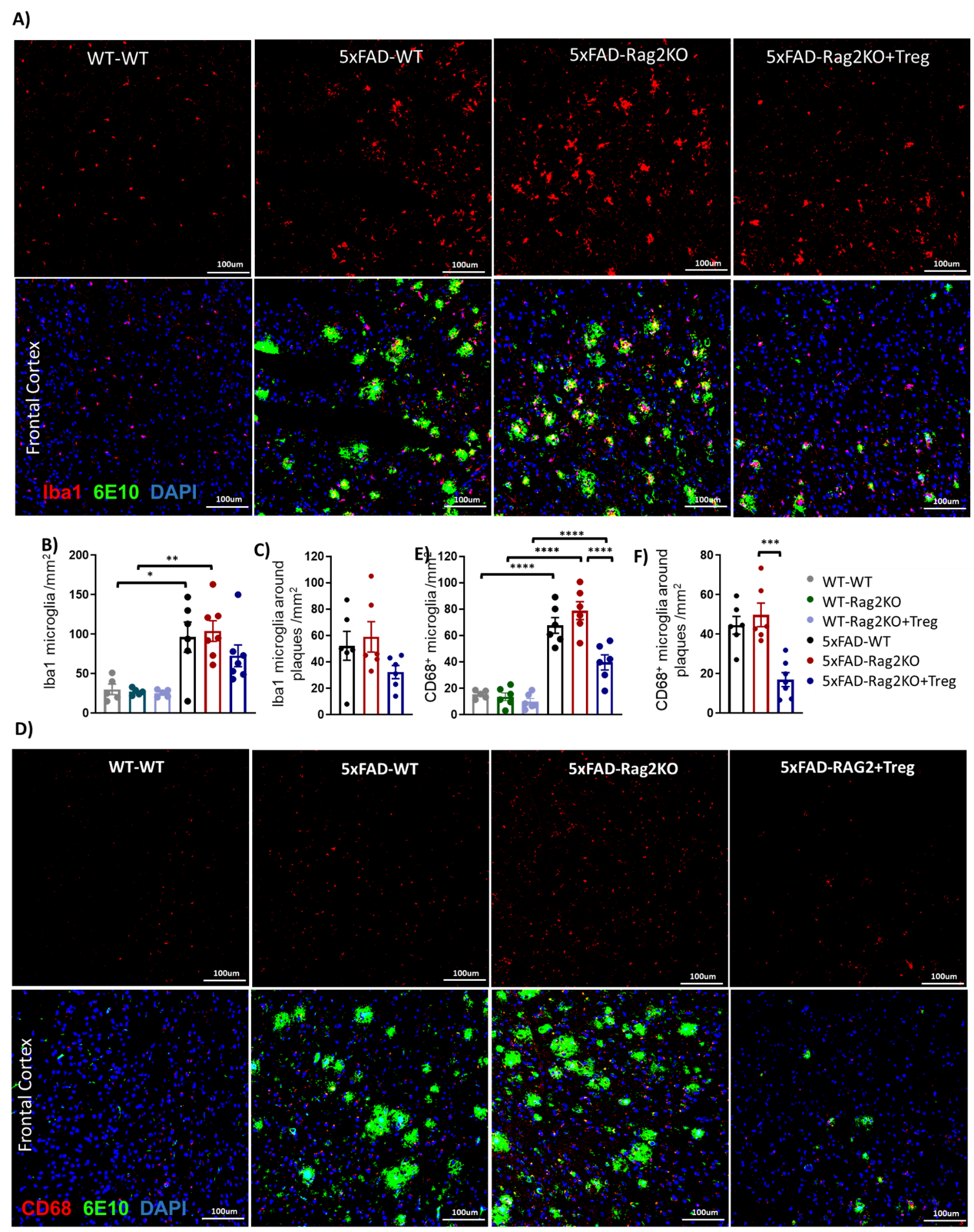


**Supplementary figure 3-** **Treg administration reduces number of activated microglia in the frontal cortex.** **(A)** Representative images of Iba1 positive-microglia (red) and 6E10-positive Aβ plaques (green) in the frontal cortex (FC) of WT-WT, 5xFAD-WT, 5xFAD-Rag2KO and Treg-treated 5xFAD-Rag2KO (n = 6 per group, sex balanced). **(B-C)** Quantification of total and plaque associated Iba+ microglia in the FC; Number of Iba1^+^microglia was increased in 5xFAD-WT and 5xFAD-Rag2KO, compared to corresponding WT groups. **(D)** Representative images of CD68-positive activated microglia (red) and 6E10-positive Aβ plaques (green) in the FC. **(E-F)** Decreased number of total and plaque-associated CD68+ microglia in 5xFAD-Rag2KO were noted following Treg administration. Numbers shown as averages ± SEM with one-way ANOVA. *P < 0.05, **P < 0.01, ***P < 0.001 and ****p< 0.0001. Scale bar, 100 μm.

**
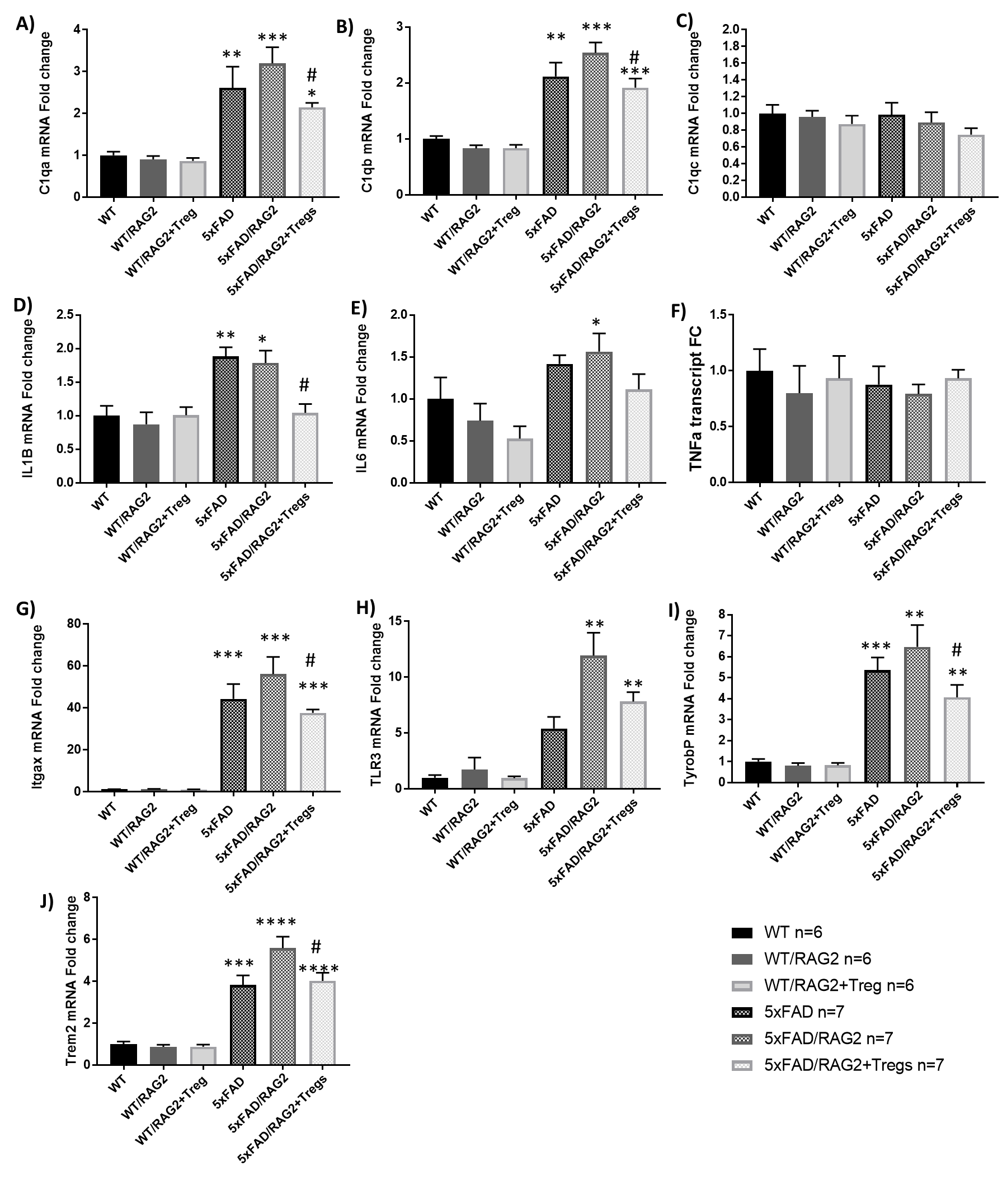
Supplementary figure 4**- Real time-PCR analysis of complement activation markers of *C1qa* (A)*, C1qb* (B)*, C1qc*(C), pro-inflammatory cytokines of *Il1b* (D)*, Il6* (E)*, Tnfa* (F), microglial activation markers of *Tyrobp* (G)*, Trem2*(H) and transmembrane protein genes of *Tlr3*(I) and *Itgax* (J) in the frontal cortex of WT-WT, WT-Rag2KO, Treg-treated WT-Rag2KO, 5xFAD-WT, 5xFAD-Rag2KO and Treg-treated 5xFAD-Rag2KO. Numbers shown as averages ± SEM with one-way ANOVA. *comparison between each AD groups versus corresponding WT ones: *P < 0.05, **P < 0.01, ***P < 0.001 and ****p< 0.0001. # p<0.05 comparison between Treg treated-5xFAD-Rag2KO versus 5xFAD-Rag2KO groups.
